# A comprehensive study of colisepticaemia progression in layer chickens applying novel tools elucidates pathogenesis and transmission of *Escherichia coli* into eggs

**DOI:** 10.1038/s41598-024-58706-3

**Published:** 2024-04-06

**Authors:** Mohamed Kamal Abdelhamid, Claudia Hess, Ivana Bilic, Martin Glösmann, Hammad Ur Rehman, Dieter Liebhart, Michael Hess, Surya Paudel

**Affiliations:** 1 Clinic for Poultry and Fish Medicine, Department for Farm Animals and Veterinary Public Health, University of Veterinary Medicine Vienna, Vienna, Austria; 2 Department of Pathology, Faculty of Veterinary Medicine, Beni-Suef University, Beni-Suef, 62511 Egypt; 3 VetCore Facility for Research/Imaging Unit, University of Veterinary Medicine Vienna, Vienna, Austria; 4 Department of Infectious Diseases and Public Health, Jockey Club College of Veterinary Medicine and Life Sciences, City University of Hong Kong, 1A-508, Block 1, To Yuen Building, 31 To Yuen Street, Kowloon, Hong Kong, SAR

**Keywords:** Chicken, Colisepticaemia, *ilux2-E. coli*, Bioluminescence, Disease stages, Egg contamination, Bacteria, Bacteriology

## Abstract

Colisepticaemia caused by avian pathogenic *Escherichia coli* (APEC) is a challenging disease due to its high economic importance in poultry, dubious pathogenesis and potential link with zoonosis and food safety. The existing in vitro studies can’t define hallmark traits of APEC isolates, suggesting a paradigm shift towards host response to understand pathogenesis. This study investigated the comprehensive pathological and microbial progression of colisepticaemia, and transmission of *E. coli* into eggs using novel tools. In total 48 hens were allocated into three groups and were inoculated intratracheally with *ilux2*-*E. coli* PA14/17480/5­/ovary (bioluminescent strain), *E. coli* PA14/17480/5­/ovary or phosphate buffered saline. Infection with both strains led to typical clinical signs and lesions of colibacillosis as in field outbreaks. Based on lung histopathology, colisepticaemia progression was divided into four disease stages as: stage I (1–3 days post infection (dpi)), stage II (6 dpi), stage III (9 dpi) and stage IV (16 dpi) that were histologically characterized by predominance of heterophils, mixed cells, pyogranuloma, and convalescence, respectively. As disease progressed, bacterial colonization in host organs also decreased, revealed by the quantification of bacterial bioluminescence, bacteriology, and quantitative immunohistochemistry. Furthermore, immunofluorescence, immunohistochemistry, and bacteria re-isolation showed that *E. coli* colonized the reproductive tract of infected hens and reached to egg yolk and albumen. In conclusion, the study provides novel insights into the pathogenesis of colisepticemia by characterizing microbial and pathological changes at different disease stages, and of the bacteria transmission to table eggs, which have serious consequences on poultry health and food safety.

## Introduction

Colibacillosis in poultry is caused by avian pathogenic *Escherichia coli* (APEC) and leads to great economic losses worldwide with the need for antimicrobial intervention^1^. The disease appears in different forms and the systemic infection called colisepticaemia is characterized by substantial polyserositis. Even though numerous in vitro studies have extensively characterized *E. coli* isolates so far, a robust genetic distinction between pathogenic and commensal isolates is still lacking^2^. Recently, a large-scale genome-wide association study (GWAS) identified complex interlinks among cohorts of genes related to nutrient uptake and host immune defense along with some novel candidate virulent genes in systemic *E. coli* isolates compared to intestinal isolates^3^. However, not all systemic isolates can be connected with a higher pathogenicity than intestinal isolates^4^. Thus, the pathogenesis of colisepticaemia remains intriguing, and it is crucial to shift the paradigm from the pathogen to host response in understanding the biology of *E. coli* infection. Previous in vivo studies of colibacillosis were primarily conducted in broilers and scarcely in layers^5^. Regarding the disease pathogenesis in layers, it is generally accepted that APEC septicemia starts with pneumonia and airsacculitis, often followed by a generalized infection^6–10^. In previous infection studies, the disease outcomes were mostly evaluated based on the assessment of gross pathology and conventional microbiological culture techniques with the latter one being complicated due to the presence of commensal *E. coli*. To date, the sequential and systematic pathogenesis of sepsis caused by *E. coli* from acute to recovery phases, including the detailed progression of microscopic changes in the host organs at defined disease stages, remains to be elucidated. Thus, further research is needed to better understand the infection biology of colisepticaemia.

Within the One Health context, APEC poses an ongoing health concern due to its genetic similarity and shared virulence genes with human extra pathogenic *E. coli* isolates including uropathogenic *E. coli*, sepsis-associated *E. coli*, and neonatal meningitis causing *E. coli*, as well as its potential to transmit antimicrobial resistant genes to humans^11,12^. Previous cross-sectional studies have shown that chicken table eggs might contain antibiotic-resistant *E. coli* isolates on eggshells and/or in egg contents^13–16^. Thus, APEC-contaminated poultry products including meat and egg are thought to be vehicles for infection by extra-intestinal *E. coli* in humans^17,18^. *E. coli* isolates from offspring and parents can have similar in vitro traits such as presence of virulence associated genes, sequence types or antimicrobial resistance profiles, suggesting vertical transmission^19–23^. However, other studies suggested pseudo vertical transmission in which *E. coli* attached to the eggshell might has the potential to penetrate inside after the eggs are laid^24^. Furthermore, *E. coli* on eggshell with shared genetic profiles of breeders and the poultry house environment, did not impact the eggs' internal contamination after disinfection^25,26^. Thus, it is still unclear whether APEC is transmitted via trans-ovarian/-oviduct transmission following colonization of reproductive organs. To fill in these knowledge gaps, the present study aimed to elucidate comprehensive pathogenesis of colisepticaemia in layers applying novel tools, bearing the potential to model respiratory sepsis in other animals as well. Additionally, the dynamics of bacterial spread, colonization of reproductive organs and egg contamination were investigated using the *ilux2*-*E. coli* PA14/17480/5/ovary bioluminescent strain enabling undoubtedly discrimination from commensal *E. coli*, based on the most updated method for the brightest expression of bioluminescence^27^. The findings provide valuable insights into the pathogenesis and transmission of APEC and its potential public health risks through table egg consumption. It also highlights the versatility of layer chickens as a model, as well as the value of bioluminescent imaging and immunohistochemistry for studying septicaemic diseases and for developing future treatment strategies.

## Results

### Clinical signs and mortality

**Figure 1:**
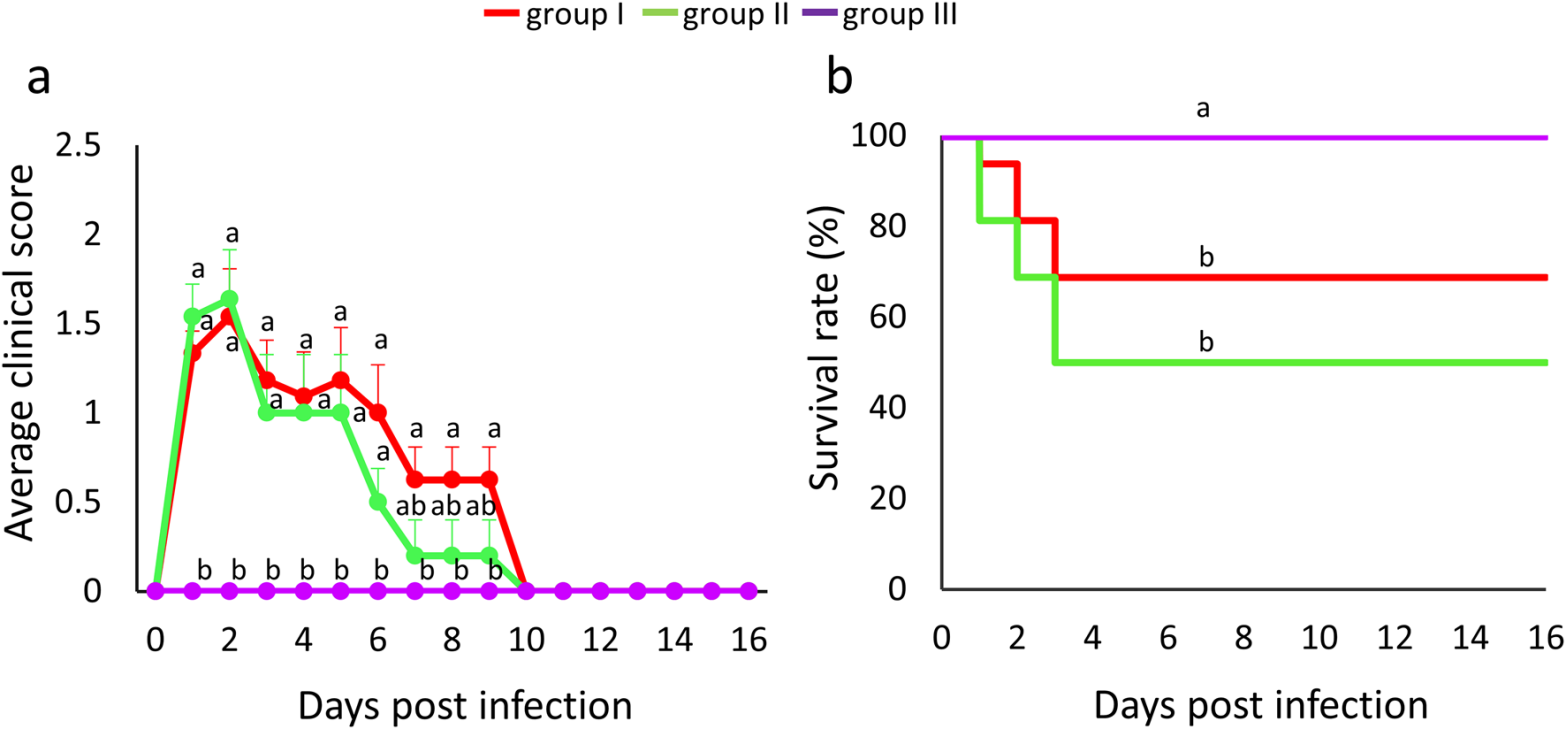
Clinical scores and mortality pattern. (**a**) Mean daily clinical scores of birds from different groups and (**b**) Kaplan–Meier curve for the survival rate after APEC infection. Group I, inoculated with *ilux2*-*E. coli* PA14/17480/5/ovary; group II, inoculated with *E. coli* PA14/17480/5/ovary; group III, negative control inoculated with PBS. Different letters denote significantly different values.

In groups I and II, clinical signs and deaths started 24 h after *E. coli* inoculation. Peak of clinical signs were observed within the first 2 days post infection (dpi) (Fig. 1a) with the average clinical signs score among the surviving birds decreasing from ≥ 0.5 at 6 dpi to zero at 10 dpi. Notably, there were no significant differences in clinical signs scores between the two infected groups during the experiment. In terms of survival rate, deaths were recorded only within 3 dpi, with 5 and 8 birds dying in groups I and II, respectively (Fig. 1b). One hen in group I reached a humane endpoint at 6 dpi and was euthanized. Chickens in the negative control group (group III) did not exhibit any clinical signs. Hence the survival rate between infected groups and the negative control group was significantly different.

### Egg production and egg weight

Prior to APEC challenge, egg production rates were similar in all groups (Supplementary Figure S1a). However, within the first 3 dpi, infected groups I and II experienced a significant decrease in egg production rates (58% and 53%, respectively) compared to the slight decrease in egg production in the negative control group III (81%). By 4–6 dpi, egg production rates in groups I and II dropped to 48% and 25%, respectively. Eventually, egg production rates returned to normal at 10 dpi, and remained steady until the end of the experiment. Remnants of shell-less egg were found at 1 dpi in each infected group, and remnants of soft-shelled egg were found in groups I and II at 12 and 5 dpi, respectively. Mean egg weight was significantly lower in both infected groups compared to the negative control group (Supplementary Figure S1b).

### Gross pathological findings

Mean macroscopic lesion scores for various organs are shown in Supplementary Figure S2a. The progression of gross lesions was characterized based on the four disease stages derived from lung histological lesions. Birds which died within 24 h of infection exhibited septicaemia, with necrosis and purulent exudate in the lung, as well as hepato-, spleno-, and renomegaly and generalized congestion. The lesion scores peaked in most of the organs at 1–3 dpi (stage I) and 6 dpi (stage II) with fibrinous exudates on organ surfaces, often mixed with yolk material on the peritoneum and reproductive organs. By 9 dpi (stage III), the lesion scores in most organs had decreased, except for the air sac, which still showed fibrinous caseous exudate. At 16 dpi (stage IV), organs appeared normal in most birds. No lesions were observed in the negative control birds throughout the experiment. In all organs, the average organ lesions scores at stage I were significantly higher in both infected groups I and II than in the uninfected group III.

### Body weight and organ to body weight ratio

The infected groups (I and II) had lower body weight than the negative control group III at 6 dpi (stage II) but recovered to normal by 9 dpi (stage III) (Supplementary Figure S2b). The ratios of liver and spleen weights to body weight were significantly higher in both infected groups at 1–3 dpi (stage I). At 16 dpi (stage IV), no significant differences between groups were observed (Supplementary Figure S2c).

### Histopathological findings

**Figure 2:**
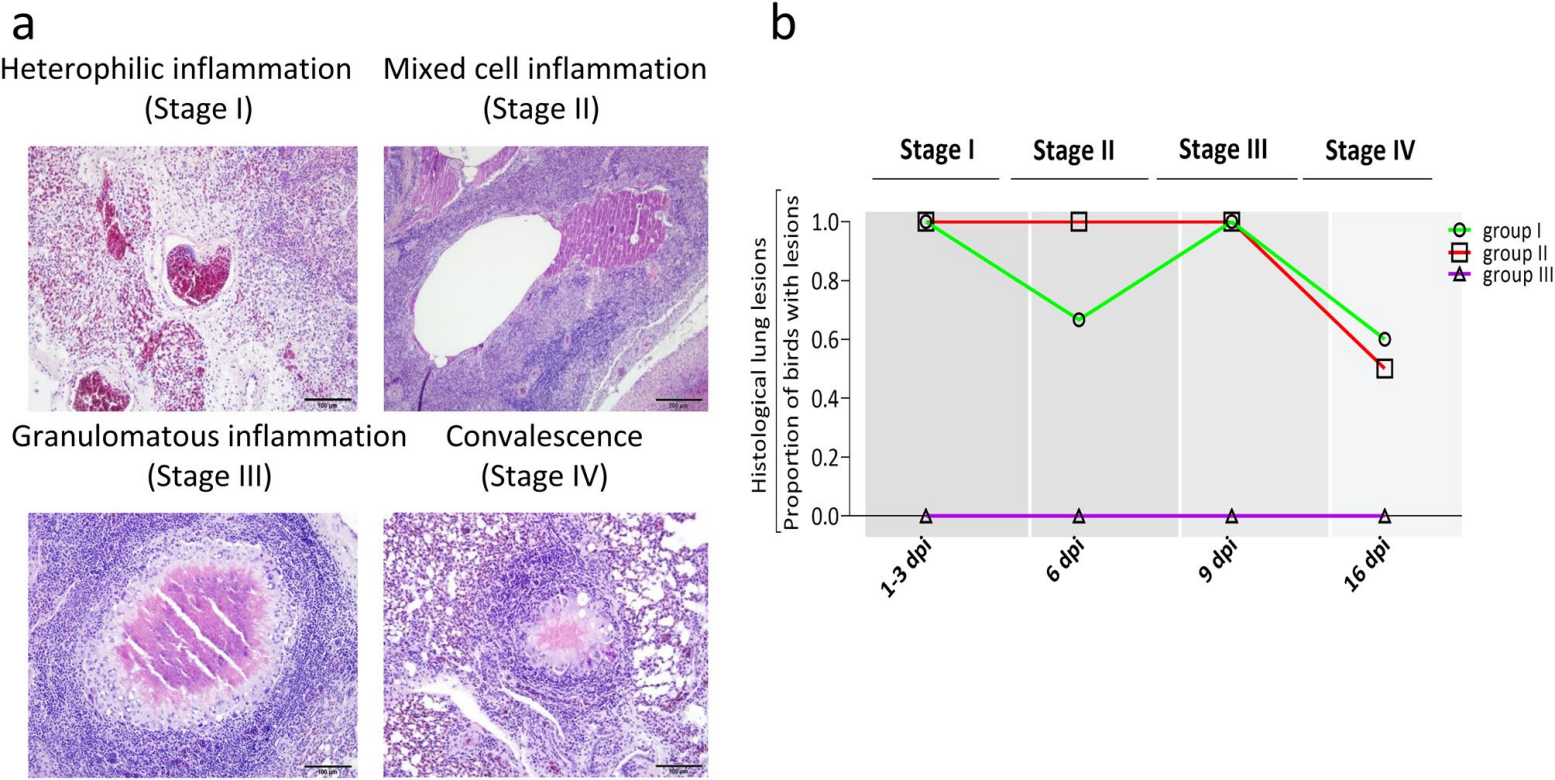
Different disease stages of colisepticaemia in experimentally infected layer birds based on the histopathological lesions in lungs. (**a**) Representative histological images of lung at each disease stage; (**b**) proportion of birds with histological lung lesions over the course of the experiment in each experimental group. Group I, inoculated with *ilux2*- *E. coli* PA14/17480/5/ovary; group II, inoculated with *E. coli* PA14/17480/5/ovary; group III, inoculated with phosphate buffered saline. dpi denotes days post infection.

Microscopically, distinct types of lung inflammation were observed at different stages of infection during progression of colibacillosis, namely heterophilic inflammation (stage I; 1–3 dpi), mixed cell inflammation (stage II; 6 dpi), pyogranulomatous inflammation (stage III; 9 dpi), and convalescence (stage IV; 16 dpi) as shown in Fig. 2a,b. Stage I was characterized by congestion, heterophilic infiltration, and necrosis in all birds that died within the first 3 dpi. Stage II featured congestion, edema, and a mixture of heterophils, macrophages, lymphocytes, and sometimes multinucleated giant cells. In stage III, congestion subsided, and granuloma appeared characterized by caseous necrosis lined by a wall of epithelioid and multinucleated giant cells surrounded by concentric rings of lymphocytic infiltrates and fibroblasts. Stage IV was characterized by complete subsiding of congestion, and remnants of pyogranulomatous reactions.

The histopathological findings in various organs are shown in Supplementary Table S1. Tracheal lesions progressed from vascular congestion and irregular sloughing of tracheal epithelium in stage I (Supplementary Figure S3a) to mild infiltration of mononuclear cells into the lamina propria at stage II and absence of lesions at the convalescent stage. Air sac lesions developed similarly to those in the lung in all stages except in convalescent stage, in which most of the killed birds still had pyogranulomas (Supplementary Figure S3b). In the heart, fibrinoheterophilic epicarditis and congested blood vessels in the myocardium and epicardium were observed at stage I, while mononuclear cell infiltration in the myocardium was observed at stages II and III (Supplementary Figure S3c). In the liver, stage I showed congestion, thickening of hepatic capsule with fibrinous exudate and heterophilic infiltration (Supplementary Figure S3d), while stages II and III showed infiltration of inflammatory cells into liver parenchyma. In the spleen, hyperaemic vascular changes, lymphoid depletion, and focal thickening of the splenic capsule with heterophilic infiltration were observed in stage I (Supplementary Figure S3e). At stages II and III, heterophilic infiltrate increased, especially in the area of red pulp persisting until the convalescent stage (Supplementary Figure S3f). In ovarian tissue, congestion of thecal layers and stroma with mild heterophilic infiltration, separation of granulosa cells from the basement membrane, and occasional deposition of fibrinous material mixed with free yolk were observed in stage I (Supplementary Figure S3g). At stage II, the lesions progressed to thickening of theca layers by edema and severe inflammatory cell infiltration mixed with bacterial colonies (Supplementary Figure S3h). At stages III and IV, most of the euthanized birds still showed inflammatory cells with yolk material and proteinaceous fluid infiltrating the ovarian stroma. Lesions in different sections of the oviduct progressed from heterophilic infiltration with proteinaceous exudate in stage I to desquamated epithelial cells and infiltration of mononuclear cells mixed with heterophils into the oviductal muscular and glandular layers, as well as intraluminal fibrinous exudate containing bacterial microcolonies in stages II and III (Supplementary Figures S1i and S3j). At the convalescent stage, most of the euthanized birds showed mononuclear cell infiltration in the glandular layers (Supplementary Figure S3k). In the brain of birds that had died, congested blood vessels with bacterial microcolonies were observed at stage I (Supplementary Figure S3l). No histological lesions were seen in organs of negative control birds.

### Bacterial bioluminescence

**Figure 3:**
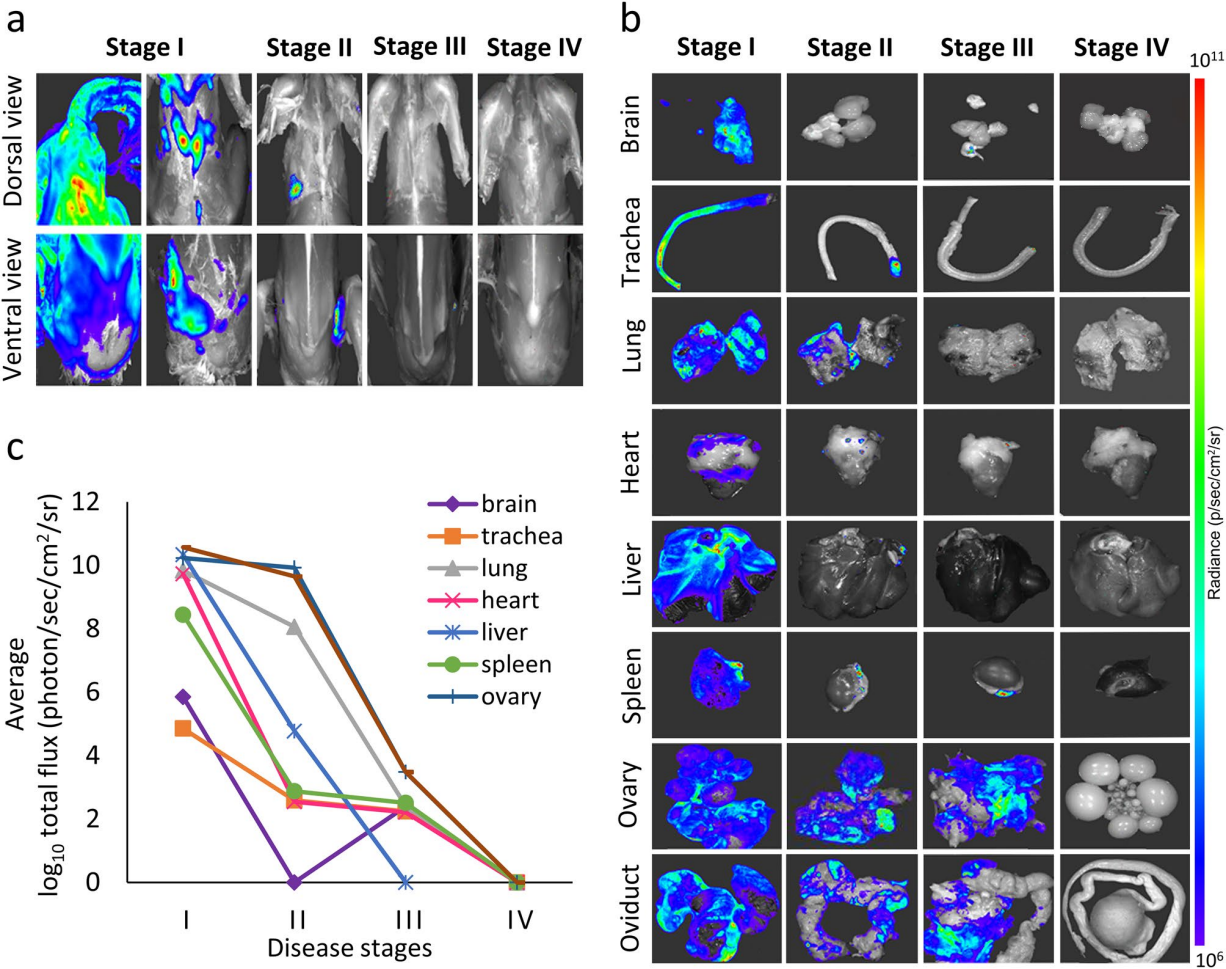
Bacterial bioluminescence at different stages of colibacillosis in experimentally infected birds. (**a**) Representative images for dorsal and ventral views of necropsied chicken layers inoculated intratracheally with *ilux2-E. coli* PA14/17480/5/ovary; (**b**) representative images from internal organs; and (**c**) quantified bioluminescent signal emitted from bioluminescent *ilux2-E. coli* PA14/17480/5/ovary in different organs.

Ex vivo bioluminescence imaging was performed on dorsal and ventral sides of whole skinned birds as shown in Fig. 3a. In general, bioluminescence signal was strong at stage I and gradually decreased until it disappeared at the convalescent stage. For internal organs, both emitted bioluminescence and average radiance were highest at stage I, decreasing in all organs except brain, where bioluminescence emission decreased until stage II and then increased again at stage III (Fig. 3b,c). At stage III, bioluminescent signal was recorded from the oviduct, ovary, lung, spleen, trachea, brain, and heart but not from the liver. At the convalescent stage, none of the organs displayed bioluminescence.

### Bacteriology

In group I, cloacal swabs from two birds were positive *for ilux2-E. coli* PA14/17480/5/ovary re-isolation at 3 and 7 dpi, while four swabs from eggshell surface, three egg white and one egg yolk sample were positive at 6 to 12 dpi. Group II had *E. coli* present on shells of two unlaid eggs at 1 dpi, and one laid egg was positive in the egg white at 9 dpi.

**Figure 4:**
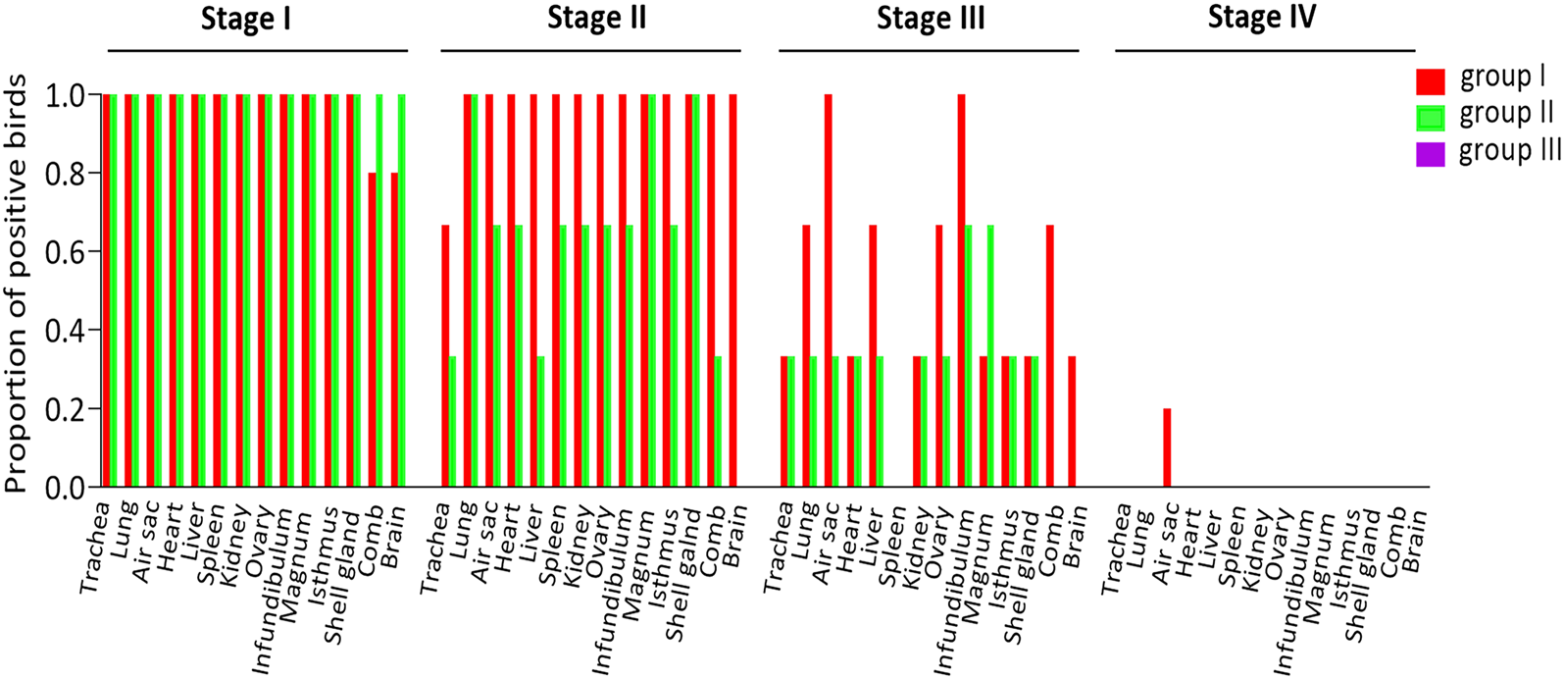
Proportion of birds positive for *E. coli* re-isolation in each experimental group at each of four disease stages. Group I, inoculated with *ilux2-E. coli* PA14/17480/5/ovary; group II, inoculated with *E. coli* PA14/17480/5/ovary; group III, inoculated with PBS.

*E. coli* was detected in multiple organs of infected birds at early disease stage, with all birds testing positive at stage I except for comb and brain in one bird from group I (Fig. 4). At stage II, all of the birds in group I were positive in every examined organ, except for the trachea from one bird while lung, magnum, and shell gland of all birds in group II were positive. The proportion of positive birds decreased in both infected groups at stage III and was zero at the convalescent stage, except for one bird in group I with a positive air sac. No bacteria were detected in organs of birds in the negative control group III.

### Immunohistochemistry (IHC) and double immunofluorescence (DIF)

**Figure 5:**
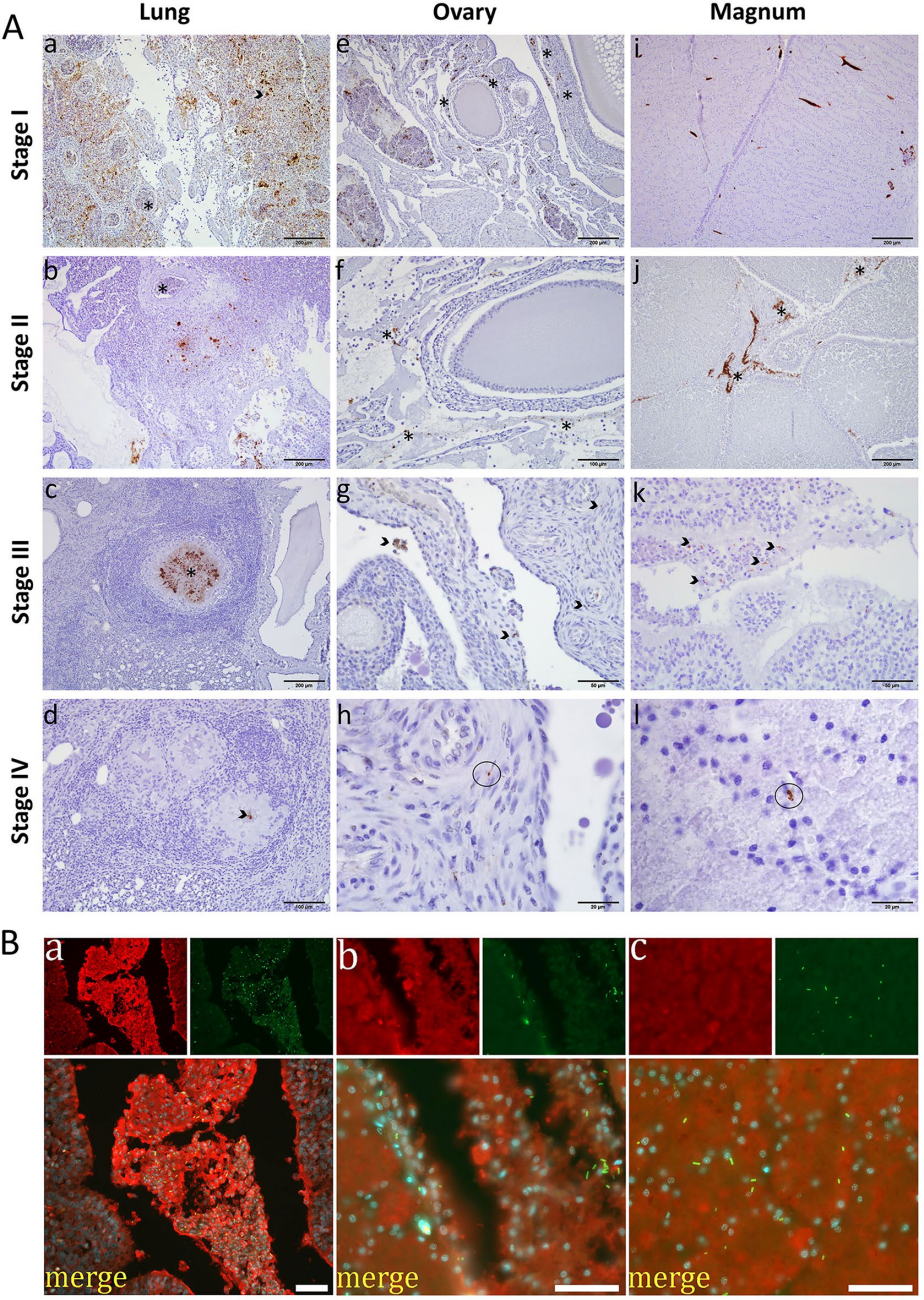
Immunostaining of *E. coli* in chicken organs*.* (**A**) Immunohistochemical detection of *E. coli* in sections of lung, ovary and oviduct magnum of group I at different time points after infection. (**a**) DAB signal in lung representing *E. coli* present in epithelium (arrowhead) and blood vessels (asterisk). (**b**) DAB positive lung stroma and blood vessel devoid of DAB signal (asterisk). (**c**) DAB positive center of a pyogranuloma (asterisk). (**d**) diminishing DAB signal at the center of a pyogranuloma (arrowhead); ovary (**e**–**h**), *E. coli* cells are widely distributed in ovarian stroma, follicle wall (asterisks) and blood vessels (**e**), proteinaceous exudate (asterisks) positive for *E. coli* antigens (**f**), arrow heads show scattered *E. coli* in ovarian stroma (**g**), circle indicates an individual *E. coli cell* in ovarian stroma (**h**); oviduct magnum (**i**–**l**), *E. coli* antigen widely distributed in whole magnum tissue (**i**), asterisks indicate positive *E. coli* antigen in secreted albumin and desquamated epithelium in the magnum lumen (**j**), arrow heads show *E. coli* antigens in degenerated mucosal epithelium of magnum (**k**), circle indicates individual *E. coli cells* in glandular epithelium cells (l). (**B**) Double fluorescence immunostaining for *E. coli* cells (green) and ovalbumin (red) in the oviduct magnum of layer hens inoculated intratracheally with APEC. Luminal egg white (**a**), mucosal epithelium (**b**), and intraglandular region (**c**). Scale bars: 40 µm.

The presence of *E. coli* in respiratory and reproductive organs was detected by IHC (Fig. 5A). The fraction of infected tissues as evidenced by DAB-staining is shown in Supplementary Table S2. The pattern of *E. coli* colonization was similar in birds from both infected groups. At stage I, *E. coli* were widely distributed in respiratory and reproductive organs. At stage II, bacterial microcolonies were still detected extensively in most organs, while at stages III and IV, they were localized in the center of pyogranulomatous reaction in the lungs and air sac, and as scattered cells in reproductive organs. *E. coli* was not detected in any of the examined organs from negative control birds. To quantify *E. coli* colonization, the proportion of DAB-stained tissue area was determined in lung, ovary, and magnum for disease stages I-IV (Supplementary Figure S4). Fractions of *E. coli* infected tissue areas were highest in lung, decreasing over time from stage I to stage IV.

In DIF-stained tissue sections from the oviduct magnum, rod-shaped *E. coli* were clearly distinguishable in both infected groups. *E. coli* were detected in the intraluminal egg white (Fig. 5Ba), in the mucosal epithelium (Fig. 5Bb), and in intraglandular tissue (Fig. 5Bc).

## Discussion

Despite the significant impact of APEC-induced septicaemia in poultry industry and the zoonotic potential of avian *E. coli* isolates, disease pathogenesis is still largely unknown. To date, neither a comprehensive description of the sequence of pathological and microbial changes during colibacillosis, nor a clear pattern of disease progression are available. Moreover, so far it has not been addressed how *E. coli* contaminates egg contents during APEC infection. The present study aims to fill this gap by systematically studying the pathology and microbial aspects of the disease and investigating how *E. coli* spreads to egg contents in a chicken layer sepsis model, which is of high significance not only for poultry health but also for food safety.

The current respiratory infection model was based on a recent trial published by our group^10^. The time points of necropsy were considered to capture the entire disease period and monitor disease severity, as well as recovery-related indicators. Along with the parent strain *E. coli* PA14/17480/5/ovary, we also used the genetically modified *ilux2*-*E. coli* PA14/17480/5/ovary strain, which allowed us to track the bacteria after inoculation in various organs and discriminate pathogenic *E. coli* from native commensal isolates. The *ilux2* operon is the brightest version of bacterial bioluminescence generated to date, which was constructed through mutations in the fatty acid reductase complex that allowed for improved utilization of fatty acids other than tetradecanoic acid^27^. In this study, the intratracheal infection of birds either with the parent or the genetically modified autonomously bioluminescent *E. coli* strain resulted in pathological lesions that were similar in type and severity, demonstrating that the integration of the *ilux2* operon does not negatively affect the virulence of parent APEC. Infected individuals showed clinical and pathological conditions similar to naturally occurring colisepticaemia. Therefore, respiratory infection was deemed the most suitable route of inoculation for the development of colisepticaemia^28^. The bacterial concentration at 10^8^ CFU/ml was commonly used in animals trials to reproduce colisepticaemia following the intratracheal inoculation^28–30^. However, in an earlier study we were unable to induce mortality and pathological lesions of comparable severity in 2 week old SPF chicks inoculated by the same route with the same *E. coli* isolate at the same bacterial concentration^31^, The difference in the disease outcome among the young SPF chicks and the commercial layers may possibly be explained by the different genetic backgrounds, host´s ages, physiological and hormonal conditions concomitant with egg laying^32^. This seems more likely than the infectious dose or the presence of maternal antibody to *E. coli*, since the same infectious dose was used at the same concentration and the maternal antibody to *E. coli* declined by day 14 of age as showed recently by us^33^. Moreover, a prior study found that there is no beneficial effect of passively received maternal antibodies on the first week mortality of chicks^34^.

The rapid development of colisepticaemia following respiratory infection, after which *E. coli* colonized all organs, evidenced by *E. coli* re-isolation and bioluminescence imaging, could potentially be attributed to the lack of free alveolar macrophages in avian lung tissue. This allowed APEC to quickly infect and spread throughout the lungs, ultimately leading to its systemic dissemination via the bloodstream^35,36^. APEC is a gram-negative bacterium with lipopolysaccharides (LPS) in its outer membrane. LPS is used as an endotoxin to induce acute phase reactions causing septic renal-hepatic injury in animal models^37^. Furthermore, LPS-induced sepsis can impair heart function or cause respiratory failure^38,39^. Based on this information, it is tempting to assume that early mortality within 24 h of infection is due to severe APEC sepsis. Lung lesions have received little attention in field studies on colibacillosis in laying chickens, unlike in broilers^40,41^. However, the lung in the current chicken layer model was the most affected organ in both challenged groups, reflecting its importance following respiratory infection with APEC. Histological changes in the lung of infected birds were regarded as reliable indicator to differentiate various stages of disease progression in comparison to clinical signs and gross lesions. For example, although the fibrinous exudate in the lung of necropsied bird was similar at 3 dpi and 6 dpi, microscopic lesions were markedly different. Specifically, at 3 dpi, there was evidence of fibrin and heterophilic inflammation, whereas at 6 dpi, a mixed cell type was present. Furthermore, some birds that appeared healthy with normal egg laying performance also had respiratory lesions, indicating yet incomplete recovery. Consequently, the actual study highlighted the importance to assess pathogenesis of colibacillosis through histopathology, which is not common in previous studies as already reviewed^5^. Therefore, most parameters in the current disease model were evaluated based on stages (heterophilic, mixed cells, pyogranulomatous, and convalescence), which were retrieved from a microscopic view of the lung lesions, providing a new perspective on the infection process.

After APEC infection, there was systemic infiltration leading to inflammation of the organs with swelling, congestion, and fibrinoheterophilic exudate. Consequently, higher organ weight indexes and decreased overall body weight in infected groups were observed, which specifically in the context of liver could be connected to the synthesis of acute phase proteins in response to inflammation^42,43^.

In this study, the lung histological changes reflected a normal sequence of the inflammatory process progressing from heterophilic inflammation (stage I) to convalescence (stage IV). A significant influx of heterophils infiltrated early after challenge, causing tissue damage that could lead to organ failure. If birds survived the acute septic stage I, the heterophils disintegrated and were phagocytosed by macrophages, leaving mononuclear cells as the dominant cell type (stage II). Over time, macrophages developed a syncytium around the necrotic area and microorganisms, forming a pyogranuloma (stage III). The defined stages aligned also to a prior investigation on hematological variables in layers after APEC infection, in which heterophilia with toxic effect in the early stage of infection indicated polymicrobial bacteremia, and then monocyte count increased at 7 dpi^44^. Similarly, monocytosis occurred after *E.coli* LPS administration in cockerels at 7 dpi^45^.

During APEC infection, immune cells release cytokines in response to bacterial invasion, causing other immune cells to be activated and recruited to the infection site. The sequential development of inflammatory reaction over the course of colisepticaemia in this study coincided with a recently published work, which showed that APEC infection led to massive secretion of proinflammatory cytokines like IL-1β, IL-6 and TNF-α expression in the lung and serum^46^. Likewise, we also showed previously that IL-1β and IL-6 can be upregulated in lung and spleen at 3 dpi^10^. Another study also showed that after APEC inoculation of CSF1R-trangenic chickens, both macrophages and heterophils were the main APEC-bearing lung phagocytes in an innate immune response dominated by heterophils. Transcriptome analysis in the same study revealed that numerous inflammatory pathways, including IL-8 and IL-6 increased after APEC inoculation^47^. These proinflammatory cytokines lead to the accumulation of associated inflammatory cells, such as heterophils and lymphocytes, either in the lung or in distant organs, causing acute inflammatory injury. Over time, the inflammatory reaction became less severe and was confined to lung tissue, showing inflammatory clusters including epithelioid and giant cells as observed in the pyogranulomatous stage (stage III). The decrease in acute inflammation over time could be attributed to increased IL-10, a regulatory cytokine that maintains immune balance and prevents excessive inflammation, as shown in a previous study in which a significant IL-10 up-regulation was observed in the lung of turkey chicks five days after APEC infection^48^. In that study, IL-10 significantly increased in the liver of birds at day 1 compared to day 28 after inoculation. This could explain why the intensity of the acute inflammation and tissue injury was milder in the liver, coinciding with an early response of IL-10, than in the lung. Finally, during the convalescent stage, pyogranulomas without the presence of bacteria or their remnants demonstrate the ability of the pyogranulomatous reaction to eliminate the microorganism from the primary site of infection as previously reported^49^.

The ex vivo bioluminescence imaging data revealed in this study are consistent with data obtained by bacterial culture, IHC and pathology, confirming a gradual decrease in bacterial load during progression of the disease from acute to convalescence stage. Whole body imaging was performed to visualize the anatomical locations of bacterial localization. Unlike imaging in mice, infected chickens with *ilux2-E. coli* did not emit a bioluminescent signal on their body surface, except after removing the feathers. This confirms the earlier observation that feathers act as a blocker^50^.

Despite the significant decrease in egg production in infected groups, the negative group also experienced a slight decrease in egg production within the first 3 days following inoculation with PBS. This may be attributed to the elevated basal plasma corticosterone level caused by fear and stress during inoculation process^51^. While our findings on egg peritonitis following intratracheal infection of birds were consistent with earlier studies^6,8,10^, we demonstrated for the first time the re-isolation of *E. coli* from egg contents (both egg white and egg yolk) in an experimental setting, providing a strong evidence for trans-ovarian/-oviduct transmission of *E. coli* from the colonized ovarian and oviduct tissues into the inner egg content. Eggshells were also tested positive for ilux2-*E. coli*, possibly from contaminated shell glands or cloaca. The transmission of avian bacterial pathogens to consumers is epidemiologically linked to the presence of pathogens in edible egg yolk and white (the current study focused on this critical issue), but contaminated eggshells can also pose an indirect threat to food safety as *E. coli* on the surface can penetrate the eggshell^24^. Alkaline pH in egg white and antibacterial substances like lysozymes and ovotransferrin are supposed to inhibit bacterial growth^52,53^. However, in the current study, four egg white and one yolk samples (out of 167 eggs) were tested positive for *E.* coli re-isolation. This could be owing to egg white's insufficient bactericidal activity^54^. In this study, we demonstrated the re-isolation of *E. coli* from the magnum, and the bacterial presence within the albumen material in magnum lumen and intra glandular regions using DIF technique, which indicated the ability of *E. coli* to contaminate egg white.

In conclusion, the elucidation of comprehensive sequential progression of APEC sepsis in this study delivered a detailed understanding of APEC pathogenesis including the disease progression pattern and highlighting the crucial role of lungs in a respiratory sepsis model. The model bears substantial potential to study sepsis related pneumonic diseases. Furthermore, the ability of *E. coli* to translocate into egg content and contaminate eggshells in infected layers bears a serious risk to public health. Therefore, it is essential to limit the burden of this potentially zoonotic pathogen in poultry and poultry products through the implementation of effective prophylactic and therapeutic intervention strategies.

## Materials and methods

### Preparation of *E. coli* inocula for infection

The *E. coli* PA14/17480/5/ovary (serotype O1:K1) was isolated from the ovary of a diseased chicken with colibacillosis, which met all the criteria to be called APEC based on in vitro and in vivo traits^9,55^. The second strain *ilux2*-*E. coli* PA14/17480/5/ovary was created by integration of the *ilux2* operon into the chromosome of *E. coli* PA14/17480/5/ovary as described before^27^. Briefly, plasmid containing *ilux2* was electroporated into *E. coli* PA14/17480/5/ovary and a population of bacterial cells carrying the plasmid was selected on LB + ampicillin agar plates at 32 °C. These cells were then grown non selectively in LB medium, and subsequently plated on LB agar at 42 °C to block the replication of the plasmid. The integration of the *ilux2* cassette in the bacterial chromosome was then validated by PCR and the whole genome sequencing of the parent and the *ilux2*-tagged strains.

For infection, both bacterial isolates were cultured, washed and their concentrations were quantified by colony forming unit (CFU) counts before and after inoculation into animals, as previously described^10^. Colonies of *ilux2*-*E. coli* PA14/17480/5/ovary isolate produce bioluminescence signals, which were imaged with an in vivo imaging system (IVIS) instrument that allow the capture of signals using the advanced camera (IVIS Lumina LT, PerkinElmer, Rodgau, Germany).

### Animals housing and experimental design

The animal trial was approved by the institutional ethics committee and the national authority according to § 8ff. of the law for Animal Experiments, Tierversuchsgesetz-TVG (License number: GZ. 2022–0.301.514). The study is reported in accordance with ARRIVE guidelines (https://arriveguidelines.org).

**Figure 6:**
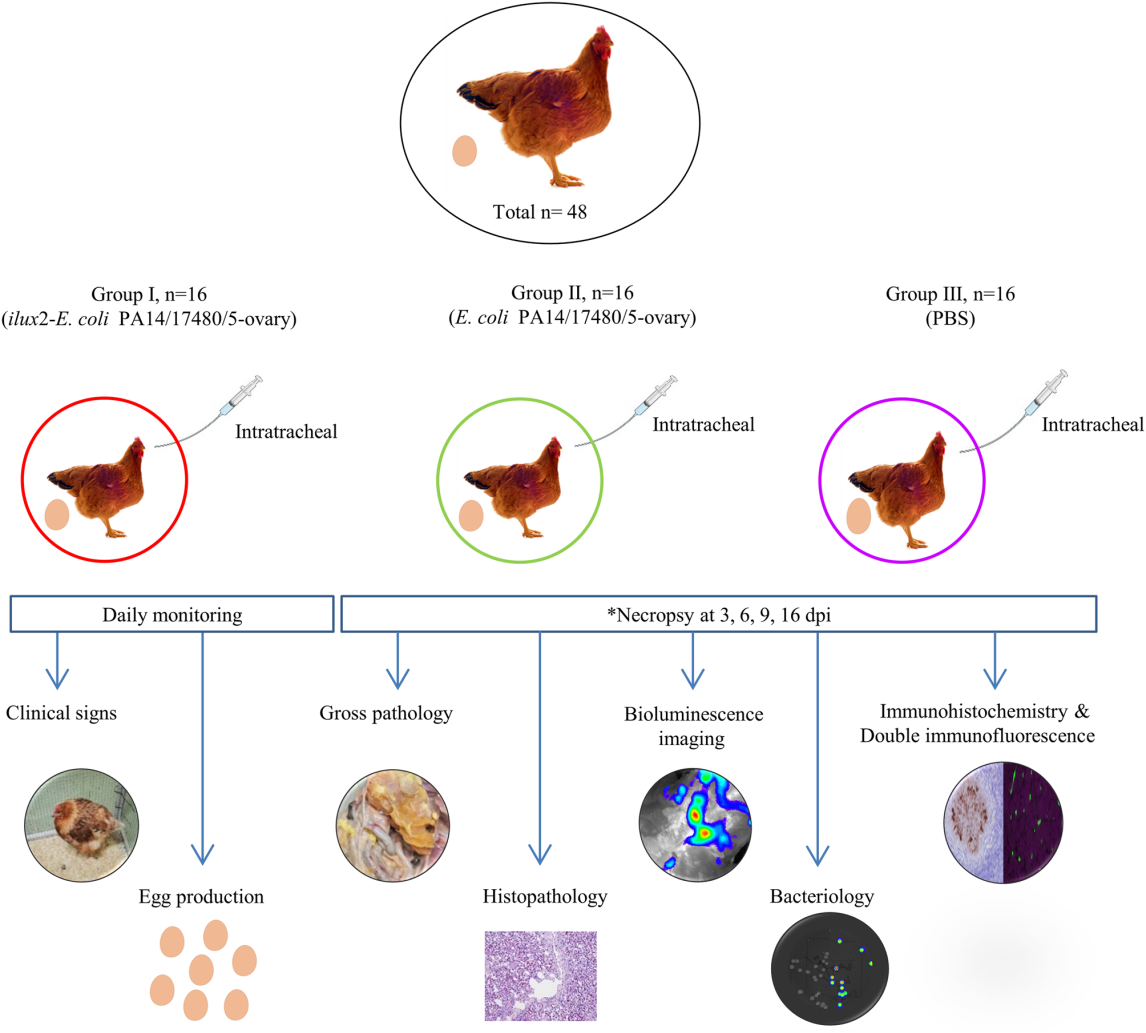
Schematic representation of the experimental design. Laying hens (n = 48) were allocated equally in three groups. Group I was infected with *ilux2-E. coli* PA14/17480/5/ovary. Group II was infected with *E. coli* PA14/17480/5/ovary. Group III was inoculated with PBS and kept as a negative control. All birds were inoculated via the intratracheal route. *n*, number of birds; *necropsy was performed on all birds that died (5 and 8 birds from groups I and II, respectively) prior to the scheduled necropsy time point; dpi, days post infection.

Forty-eight 18-week-old commercial layer pullets (Schropper GmbH, Gloggnitz, Austria) were randomly divided into three groups (Fig. 6), each containing 16 birds, and housed separately in a negatively pressured room on deep litter. The air pressure and light program were optimally controlled, and feed and water were provided ad libitum. The number of eggs laid by each group of hens was recorded to calculate egg production performance (number of eggs laid/number of birds), and each egg was weighed. At 26 weeks of age, hens in group I, II and III were intratracheally inoculated with 1 ml of 3.1 × 10^8^ CFU/ml of *ilux2*-*E. coli* PA14/17480/5/ovary, 3.4 × 10^8^ CFU/ml of *E. coli* PA14/17480/5/ovary and phosphate buffered saline (PBS), respectively. All birds were monitored daily for clinical signs according to the scoring scheme presented in Supplementary Table S3. Necropsy and sampling were performed at 3, 6, 9, and 16 dpi. Euthanasia was performed by intravenous administration of thiopental (Medicamentum pharma GmbH, Austria) followed by bleeding to death. The macroscopic lesions of the affected organs were scored during necropsy using a scoring system published earlier^10,30^ with some modifications (Supplementary Table S4).

### Body weight and organ weight to body weight ratio

Total body weight and the weights of liver and spleen were recorded during necropsy. The organ to body weight ratio was calculated as: (weight of organ/body weight) × 100.

### Histopathology

Tissue from lung, trachea, air sac, liver, spleen, ovary, and oviduct was collected during necropsy. Tissue samples were fixed in 10% neutral buffered formalin, embedded in paraffin wax, sectioned into 5 μm slices, and stained with haematoxylin and eosin following a standard protocol for microscopic examinations.

### Bioluminescence imaging and quantification

All birds from group I, both those that had died acutely and those euthanized at specific killing points, were deskinned and the whole birds were imaged from dorsal and ventral. After that, necropsy was performed to excise internal organs (brain, trachea, lung, heart, liver, spleen, ovary, and oviduct) and the organs were placed separately on Petri dishes to visualize the signal emitted from the surface of each organ using in vivo imaging system instrument with a binning of 16 (large) and a f/stop of 1 (IVIS Lumina LT, PerkinElmer, Rodgau, Germany). The bioluminescent signal emitted by the *ilux*2-*E. coli* PA14/17480/5/ovary was analyzed using the Living Image software (version 4.5.5; PerkinElmer). Images were depicted in pseudo-colors. For quantification, photon-flux (photons/second/cm^2^/steradian) was measured from one to several free-drawn regions of interest (ROI) per optical image^56^.

### Bacteriology

Birds in group I were swabbed for cloacal shedding of *ilux2-E. coli* PA14/17480/5/ovary before and, twice a week, after infection. Eggshell surfaces of freshly laid eggs were also swabbed using a sterile cotton swab soaked in LB broth. Swabs were then incubated overnight at 37 °C in 4 ml LB broth. If bioluminescence signals were detected, cultures were further streaked on LB agar for bacterial re-isolation. Unlaid eggs in the shell gland of birds from groups II and III were swabbed on their surfaces during necropsy. For re-isolation of *E. coli* from egg internal contents, each egg from all groups was dipped in 70% ethanol for 1 min. The egg contents (albumen and yolk) were then individually emptied into sterile tubes, homogenized, and 1 ml of the sample was mixed with 4 ml of LB broth and incubated at 37 °C for 24 h. For bacterial re-isolation from trachea, lung, air sac, heart, liver, spleen, ovary, infundibulum, magnum, isthmus, shell gland, comb, and brain of the necropsied birds, organs were directly streaked on LB agar plates and incubated at 37 °C. All samples from group I, either LB broth inoculated by swabs or streaked agar plates were visualized under the IVIS after the overnight incubation to detect the bioluminescent signal emitted from the *ilux*2-tagged *E. coli* isolate.

### Immunohistochemistry and double immunofluorescence

Paraffin embedded tissue sections of the trachea, lung, air sacs, ovary, and oviduct from all birds were processed for IHC using an anti-*E. coli* LPS antibody (2D7/1, ab35654, Abcam) following a previously published protocol^57^. For signal visualization, the VECTASTAIN ABC kit (vector Laboratories) was used with DAB as a substrate. Counter staining was performed with Mayer’s hematoxylin (Merck KGaA, Daemstadt, Germany) and the color reaction was observed under a microscope. For quantification of the DAB-stained *E. coli* in the lung, ovary and magnum, the slides from groups I and III were scanned using a 20X objective lens on a Sideview VS200 (Evident, Waltham, MA, USA) and the proportion of tissue area positive for DAB was quantified using QuPath (version v0.4.3; https://qupath.github.io/).

A double immunofluorescence protocol was established for the localization of *E. coli* in the egg white, either in the lumen or in glands of the magnum portion of the oviduct. Briefly, magnum sections were deparaffinized, rehydrated, and blocked with normal goat serum. Sections were incubated in a humid chamber at 4 °C overnight with a mixture of monoclonal mouse anti-*E. coli* LPS (2D7/1) (ab35654, Abcam) and polyclonal rabbit anti-chicken ovalbumin (NB600-922, Novus) at a dilution of 1:500. After washing in PBS, sections were incubated with secondary antibodies (Alexa Fluor™ 488 goat anti-mouse IgG (H + L), 1:1000, and Alexa Fluor 568™ goat anti-rabbit IgG (H + L), 1:500, Invitrogen) for 60 min at room temperature, stained with DAPI (D9542, Sigma-Aldrich, 1:1000), mounted and imaged using a Zeiss Axio Imager.Z2 (Zeiss, Oberkochen, Germany).

### Statistical analysis

The statistical analysis was performed using SPSS (IBM® SPSS® version 27; IBM Cooperation, New York, United States). To test for normality, the Shapiro–Wilk test was employed. For data that followed a normal distribution, one-way analysis of variance (ANOVA) followed by post-hoc Tukey's multiple comparison test was used to evaluate differences between groups. In cases where the data did not follow a normal distribution, pairwise comparisons were conducted using the Mann–Whitney *U* test as a nonparametric approach. The data were presented as mean ± SEM, and statistical significance was considered at *P* ≤ 0.05.

### Supplementary Information

Supplementary Figure S1.

Supplementary Figure S2.

Supplementary Figure S3.

Supplementary Figure S4.

Supplementary Information 5.

## Data Availability

The data supporting the findings of this study are available in the manuscript or its supplementary materials.
