# Supplementary figures and images for: A comprehensive study of colisepticaemia progression in layer chickens applying novel tools elucidates pathogenesis and transmission of Escherichia coli into eggs
Source: Sci Rep. 2024 Apr 6;14:8111. doi: 10.1038/s41598-024-58706-3 (PMC10998890; doi:10.1038/s41598-024-58706-3)

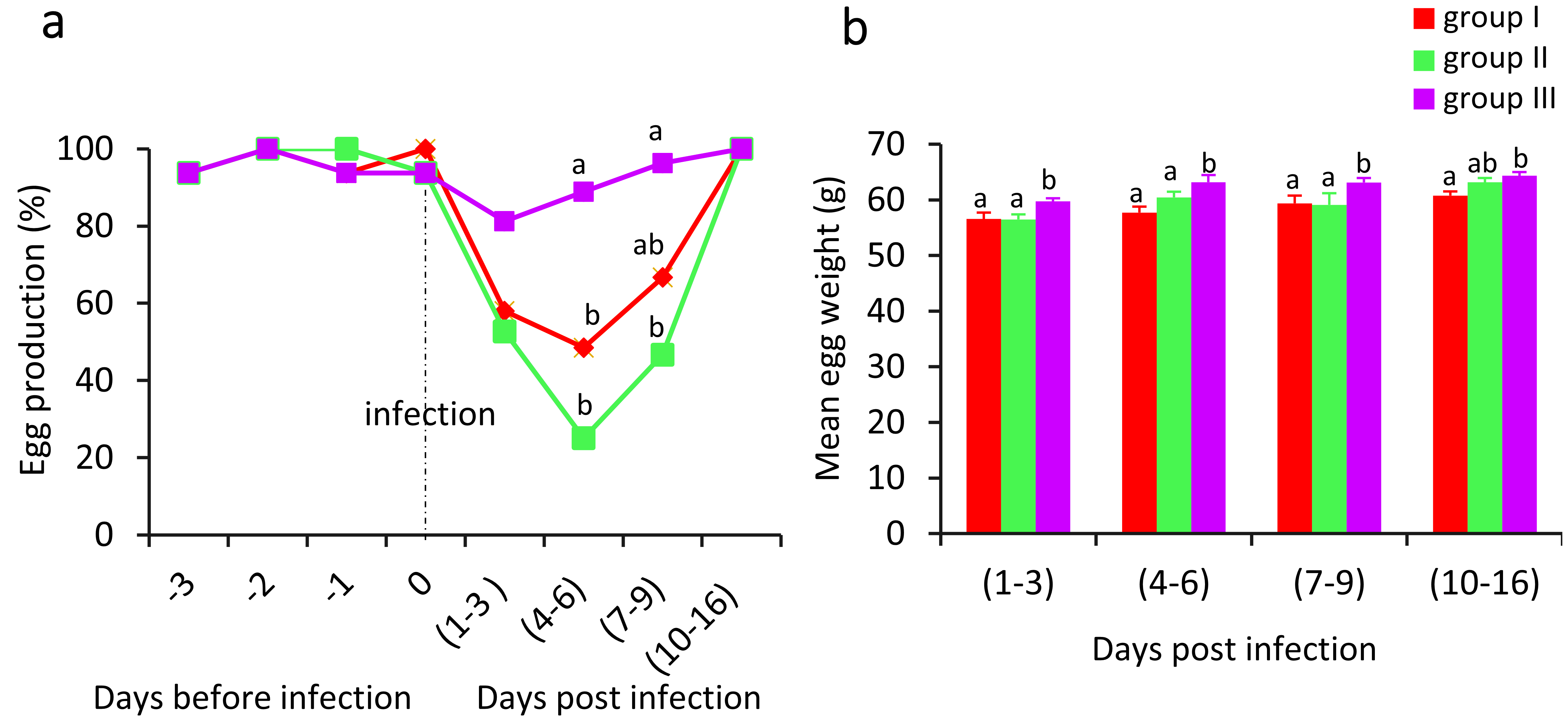

Supplement: Supplementary file 1 — Supplementary Figure S1. [file 41598_2024_58706_MOESM1_ESM.tif]

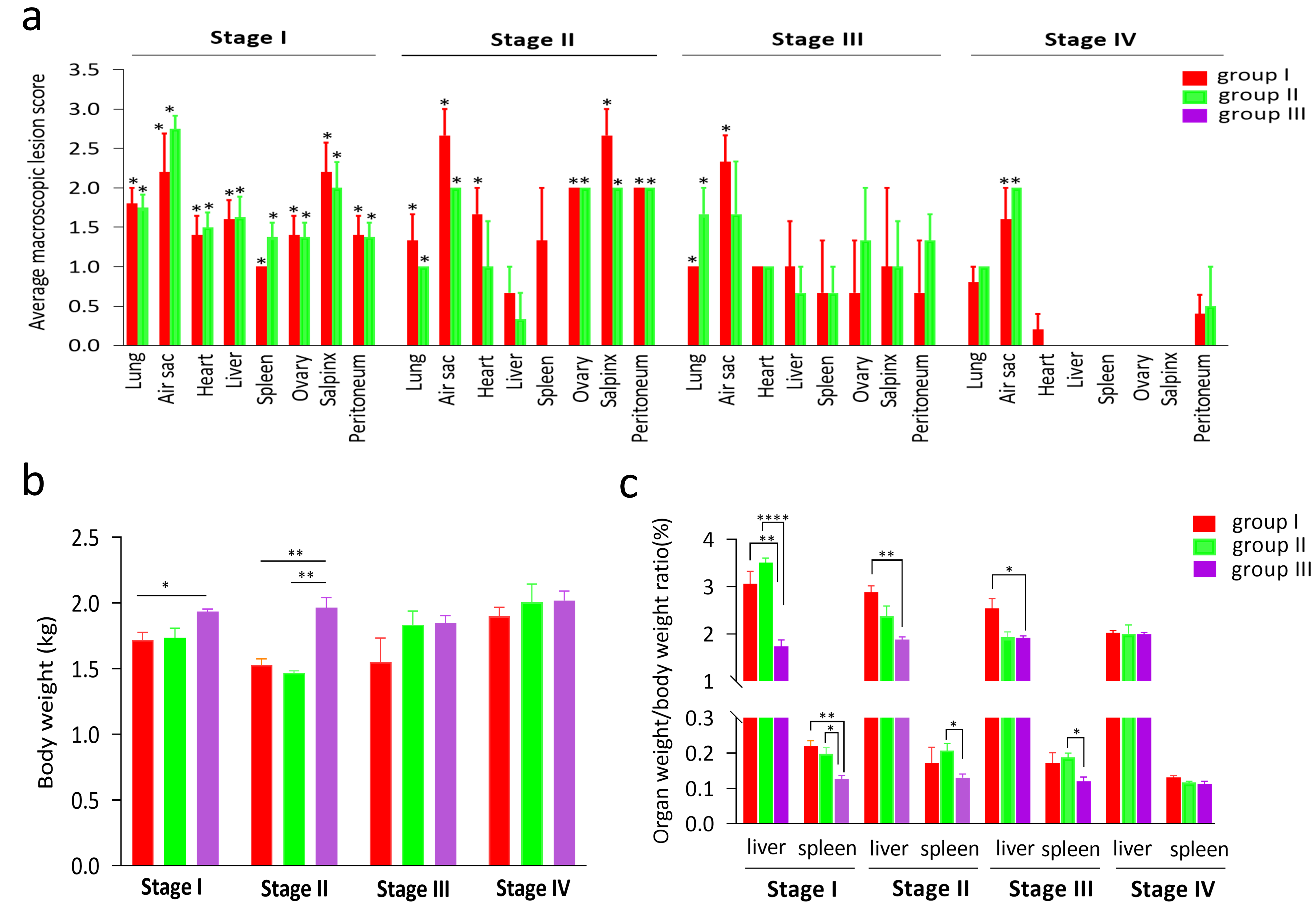

Supplement: Supplementary file 2 — Supplementary Figure S2. [file 41598_2024_58706_MOESM2_ESM.tif]

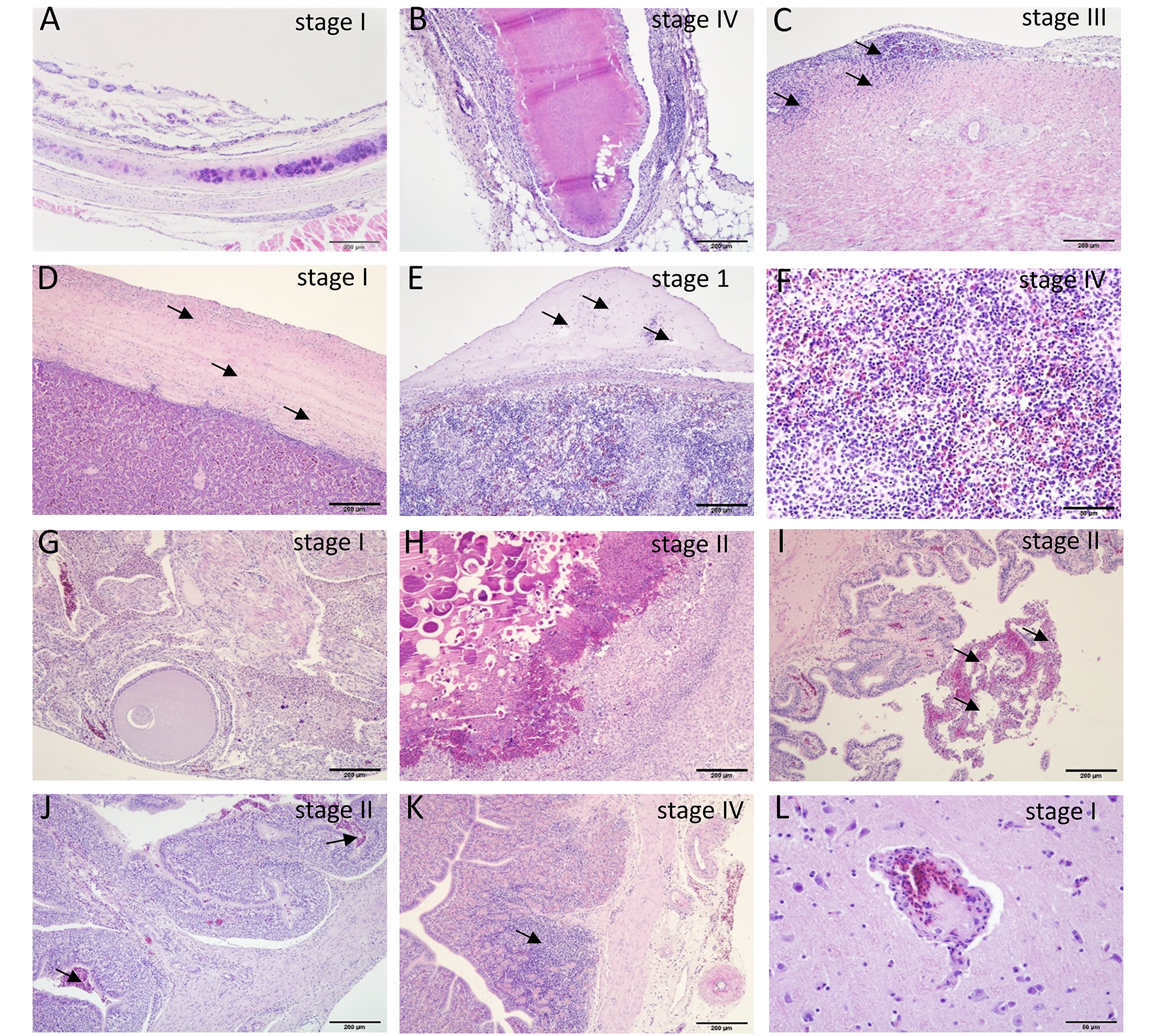

Supplement: Supplementary file 3 — Supplementary Figure S3. [file 41598_2024_58706_MOESM3_ESM.tif]

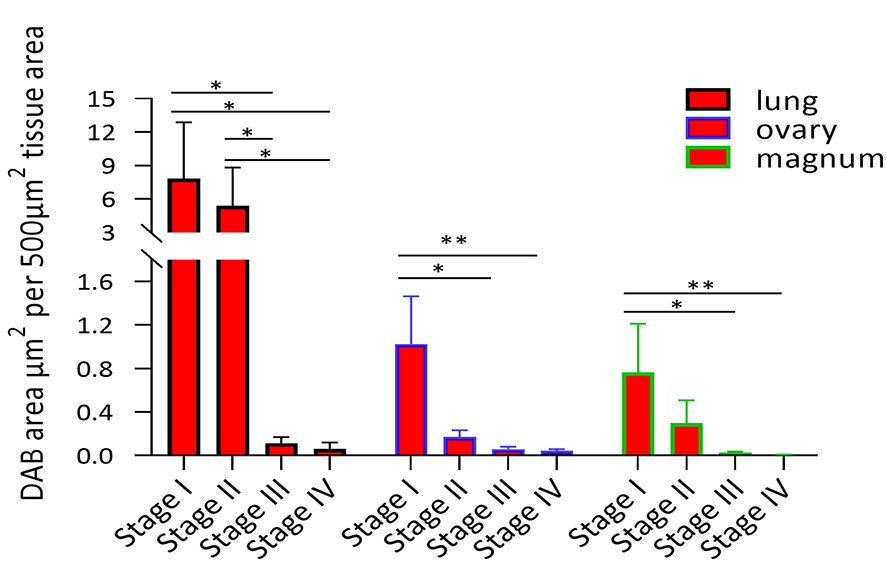

Supplement: Supplementary file 4 — Supplementary Figure S4. [file 41598_2024_58706_MOESM4_ESM.tif]
