# Supplementary material for: A comprehensive study of colisepticaemia progression in layer chickens applying novel tools elucidates pathogenesis and transmission of Escherichia coli into eggs
Source: Sci Rep. 2024 Apr 6;14:8111. doi: 10.1038/s41598-024-58706-3 (PMC10998890; doi:10.1038/s41598-024-58706-3)
Supplement: Supplementary file 5 — Supplementary Information 5. [file 41598_2024_58706_MOESM5_ESM.docx]

# Supplementary information

Supplementary Figure S1. Egg production performance and egg weight. (a) Average percentage of egg production, and (b) average weight of eggs laid by birds in groups I, II and III, which were inoculated with *ilux2-E. coli* PA14/17480/5-ovary, *E. coli* PA14/17480/5-ovary and PBS, respectively. Different letters denote significantly different values.

Supplementary Figure S2. Macroscopic lesions, body weight and organ to body weight ratio at different disease stages. (a) Average macroscopic lesion scores in different organs. *Significantly different from the negative control group; (b) average body weight and (c) liver and spleen weight to body weight ratio. Birds necropsied at different time points were used to calculate these values, thus at 1-3 dpi (stage I) average was obtained from 5, 8 and 4 birds in groups I, II and III, respectively. Likewise, at 6 dpi (stage II) and 9 dpi (stage III) 3 birds from each group were necropsied. At 16 dpi (stage IV), 5, 2 and 6 birds were killed for necropsy in groups I, II and III, respectively. Birds in groups I, II and III were inoculated with ilux2-E. coli PA14/17480/5-ovary, *E. coli* PA14/17480/5-ovary and phosphate buffered saline, respectively. Asterisks in b and c represent statistical significance (*p ≤ 0.05; **p ≤ 0.01; ****p ≤ 0.0001)

Supplementary Figure S3. Histopathological lesions in different organs of laying hens experimentally inoculated with APEC via the intratracheal route. (a) Trachea, tracheitis with partly sloughing of mucosal epithelium; (b) air sac, pyogranulomatous airsacculitis characterized by a large number of degranulating heterophils mixed with bacteria and fibrin, which in turn are surrounded by multi-nucleated giant cells, epithelioid cells, lymphocytes, and fibrosis; (c) heart, cellular infiltrate in the epicardium extended to the myocardium (arrows); (d) liver, a thick layer of fibrinosuppurative exudate overlying the capsule of liver (arrows) along with congestion of hepatic sinusoids; (e) spleen, a layer of fibrinosuppurative exudate overlying the splenic capsule (arrows) along with congestion and multi focal necrotic areas; (f) spleen, substantial heterophilic infiltration; (g) ovary, heterophilic infiltration in ovarian stroma with mature follicle showing separation of granulosa cells from its basement membrane; (h) ovarian follicle, disruption in layers with yolk globules containing bacterial colonies mixed with infiltrating heterophils; (i) oviduct (infundibulum), luminal fibrinoheterophilic material mixed with desquamated mucosal epithelium (arrows); (j) oviduct (isthmus), luminal masses of degenerated heterophils, and albuminous material (arrows) along with glandular cellular infiltration; (k) oviduct (magnum), glandular cellular infiltration (arrow); and (l) brain, congested blood vessel containing bacteria

Supplementary Figure S4. Quantitative analysis of *E. coli* positive DAB-stained areas in lung, ovary, and oviduct magnum of birds from group I during each stage of disease. Asterisks represent statistical significance (*p ≤ 0.05; **p ≤ 0.01).

| **Supplementary Table S1**. Histological lesions in different tissues of *E. coli* infected birds at each disease stage. | | | | | | | | | | |
| --- | --- | --- | --- | --- | --- | --- | --- | --- | --- | --- |
| **Lesions description** | | Stage I | | | Stage II | | Stage III | | Stage IV | |
|  |  | group I  (n=5) | | group II  (n=8) | group I  (n=3) | group II  (n=3) | group I  (n=3) | group II  (n=3) | group I  (n=5) | group II  (n=2) |
| **trachea** | | | | | | | | | | |
| - irregular sloughing of the tracheal epithelium as well as vascular congestion in the lamina propria | | | 5^*^ | 8 | 3 | 2 | 0 | 0 | 0 | 0 |
| - mild infiltration of mononuclear cells into the lamina propria | | | 0 | 0 | 0 | 1 | 3 | 2 | 0 | 0 |
| - lesion free | | | 0 | 0 | 0 | 0 | 0 | 1 | 5 | 2 |
| **air sac** | | |  |  |  |  |  |  |  |  |
| - congested blood vessels with heterophilic infiltration which underwent necrosis in some cases | | | 5 | 8 | 0 | 0 | 0 | 0 | 0 | 0 |
| - still featured congestion and edema along with mononuclear cell infiltration | | | 0 | 0 | 2 | 3 | 0 | 0 | 0 | 0 |
| - pyogranulomatous reaction | | | 0 | 0 | 1 | 0 | 3 | 3 | 4 | 2 |
| - lesion free | | | 0 | 0 | 0 | 0 | 0 | 0 | 1 | 0 |
| **heart** | | |  |  |  |  |  |  |  |  |
| - general congestion of blood vessels in epi and myocardium | | | 3 | 3 | 1 | 1 | 0 | 0 | 0 | 0 |
| - thickening of the pericardium with heterophilic infiltration intermixed with fibrinous exudate | | | 2 | 5 | 0 | 0 | 0 | 0 | 0 | 0 |
| - epicardium was infiltrated by mononuclear cells that can extend to the myocardium | | | 0 | 0 | 1 | 0 | 2 | 2 | 0 | 0 |
| - lesion free | | | 0 | 0 | 1 | 2 | 1 | 1 | 5 | 2 |
| **liver** | | |  |  |  |  |  |  |  |  |
| - distention of blood vessels and hepatic sinusoids with blood | | | 4 | 4 | 1 | 0 | 0 | 0 | 0 | 0 |
| - focal thickening of the hepatic capsule with fibrinous exudate and heterophilic infiltration | | | 1 | 4 | 0 | 0 | 1 | 0 | 0 | 0 |
| - liver parenchyma was infiltrated by inflammatory cells | | | 0 | 0 | 1 | 0 | 0 | 2 | 0 | 0 |
| - lesion free | | | 0 | 0 | 1 | 3 | 2 | 1 | 5 | 2 |
| **spleen** | | |  |  |  |  |  |  |  |  |
| - hyperaemic vascular changes associated with lymphoid depletion | | | 3 | 5 | 0 | 0 | 0 | 0 | 0 | 0 |
| - focal thickening of the splenic capsule with heterophilic infiltration | | | 2 | 3 | 0 | 0 | 0 | 0 | 1 | 0 |
| - increased infiltration with heterophils, especially in the area of red pulp | | | 0 | 0 | 3 | 3 | 3 | 3 | 4 | 2 |
| **ovary** | | |  |  |  |  |  |  |  |  |
| - general congestion of thecal layers and ovarian stroma blood vessels, mild heterophilic infiltration, and separation of granulosa cells from the basement membrane | | | 3 | 2 | 0 | 0 | 0 | 0 | 0 | 0 |
| - fibrinous material occasionally mixed with free yolk material adhering to theca externa layers | | | 2 | 6 | 0 | 0 | 0 | 1 | 0 | 0 |
| - thickening of thecal layers with edema and severe infiltration of inflammatory cells | | | 0 | 0 | 3 | 3 | 1 | 0 | 0 | 0 |
| - ovarian follicles free of lesions; however, the ovarian stroma still had inflammatory cells, yolk material, and proteinaceous fluid infiltrating. | | | 0 | 0 | 0 | 0 | 2 | 2 | 3 | 1 |
| - lesion free | | | 0 | 0 | 0 | 0 | 0 | 0 | 2 | 1 |
| **oviduct** | | |  |  |  |  |  |  |  |  |
| - marked congestion of serosal and mucosal layers with degeneration and necrosis of surface epithelium | | | 4 | 3 | 0 | 0 | 0 | 0 | 0 | 0 |
| - deposition of Proteinaceous exudate with heterophilic infiltration on the serosal surface of the oviduct | | | 1 | 5 | 2 | 0 | 2 | 0 | 1 | 0 |
| - infiltration of mononuclear cells mixed with heterophils into the muscular and glandular layers of the oviduct along with desquamated epithelial cells, fibrinous exudate with bacterial microcolonies in the lumen | | | 0 | 0 | 1 | 3 | 1 | 2 | 0 | 0 |
| - mononuclear cell infiltration in the glandular layers | | | 0 | 0 | 0 | 0 | 0 | 1 | 4 | 1 |
| - lesion free | | | 0 | 0 | 0 | 0 | 0 | 0 | 0 | 1 |
| dpi: days post infection; ^*^number of birds positive for the lesion during each of the proposed stages, i.e., stage I (heterophilic inflammation), stage II (mixed inflammation), stage III (pyogranulomatous inflammation), stage IV (convalescence). group I, *ilux2-E. coli* PA14/17480/5­-ovary inoculated birds; group II, *E. coli* PA14/17480/5­-ovary inoculated birds; group III, negative control | | | | | | | | | | |

**Supplementary Table S2**. Immunohistochemical detection of *E. coli* in respiratory and reproductive organs at the four disease stages.

| organ | group I (n= 16) | group II (n= 16) | group III (n= 16) |
| --- | --- | --- | --- |
| **trachea** | | | |
| stage I | ^*^5/5 | 8/8 | 0/4 |
| stage II | 3/3 | 1/3 | 0/3 |
| stage III | 1/3 | 0/3 | 0/3 |
| stage IV | 0/5 | 0/2 | 0/6 |
| **lung** | | | |
| stage I | 5/5 | 8/8 | 0/4 |
| stage II | 3/3 | 2/3 | 0/3 |
| stage III | 3/3 | 2/3 | 0/3 |
| stage IV | 1/5 | 1/2 | 0/6 |
| **air sac** | | | |
| stage I | 5/5 | 8/8 | 0/4 |
| stage II | 3/3 | 2/3 | 0/3 |
| stage III | 3/3 | 2/3 | 0/3 |
| stage IV | 1/5 | 1/2 | 0/6 |
| **ovary** | | | |
| stage I | 5/5 | 8/8 | 0/4 |
| stage II | 3/3 | 3/3 | 0/3 |
| stage III | 3/3 | 2/3 | 0/3 |
| stage IV | 3/5 | 1/2 | 0/6 |
| **oviduct** | | | |
| stage I | 5/5 | 8/8 | 0/4 |
| stage II | 3/3 | 3/3 | 0/3 |
| stage III | 3/3 | 2/3 | 0/3 |
| stage IV | 3/5 | 1/2 | 0/6 |
| group I, *ilux2*-*E. coli* PA14/17480/5­-ovary inoculated birds; group II, *E. coli* PA14/17480/5­-ovary inoculated birds; group III, negative control. ^*^Number of DAB-positive individuals /number of examined individuals. Data shown for five different tissues and all experimental groups. | | | |

| Supplementary Table S3. Clinical scoring scheme | |
| --- | --- |
| score | clinical signs |
| 0 | active with no clinical symptoms |
| 1 | slightly weak, dropping wings and depressed |
| 2 | weak and ruffled feathers, reluctant to move, apathy |
| 3 | animal unable to move or stand, eyes closed and intensified breathing |

| Supplementary Table S4. Macroscopic lesion scoring scheme | | |
| --- | --- | --- |
| Organ | Score | Lesion |
| Lung | 0 | no pathological changes |
|  | 1 | lesions are in ≤ 1/2 of the of the lung (uni-or bilateral) |
|  | 2 | lesions are in > 1/2 of the of the lung (uni-or bilateral) |
| Air sac | 0 | no pathological changes |
|  | 1 | slightly opaque and/or thickened membranes |
|  | 2 | moderately thickened air sac with small amount of fibrin |
|  | 3 | severely thickened air sac with massive amount of fibrin |
| Heart | 0 | no pathological changes |
|  | 1 | opacity with lack of transparency of pericardium |
|  | 2 | thickened pericardium with marked pericarditis |
| Liver | 0 | no pathological changes |
|  | 1 | congested, enlarged and friable liver |
|  | 2 | slight amount of fibrin on liver surface |
|  | 3 | massive amount of fibrin covering liver surface |
| Spleen | 0 | no pathological changes |
|  | 1 | enlarged and congested spleen |
|  | 2 | fibrin deposits on splenic capsule |
| Ovary | 0 | no pathological changes |
|  | 1 | oophoritis accompanied by congested follicles and/ or presence of purulent exudative deposits |
|  | 2 | oophoritis accompanied by follicle rapture, regression, and deformities in ovarian follicles |
| Salpinx | 0 | no pathological changes |
|  | 1 | salpingitis with congested wall and /or exudative deposits |
|  | 2 | firmly clumped oviduct |
|  | 3 | salpingitis accompanied by masses of caseous and inspissate yolk materials |
| Peritoneum | 0 | no pathological changes |
|  | 1 | slight edematous exudates and/or fibrin spots inside the peritoneum |
|  | 2 | markedly edematous with fibrinous masses inside the peritoneum |
